# Endophytic *Penicillium funiculosum* LHL06 secretes gibberellin that reprograms *Glycine max* L. growth during copper stress

**DOI:** 10.1186/1471-2229-13-86

**Published:** 2013-05-31

**Authors:** Abdul Latif Khan, In-Jung Lee

**Affiliations:** 1 Department of Biological Sciences & Chemistry, University of Nizwa, Nizwa 616, Sultanate of Oman; 2 School of Applied Biosciences, College of Agriculture and Life Sciences, Kyungpook National University, Daegu, 701-702, Republic of Korea; 3 Kohat University of Science & Technology, Kohat, Pakistan

**Keywords:** *Glycine max L*, Root essential nutrients, Endophytism, Copper stress

## Abstract

**Background:**

Heavy metal pollution in crop fields is one of the major issues in sustainable agriculture production. To improve crop growth and reduce the toxic effects of metals is an ideal strategy. Understanding the resilience of gibberellins producing endophytic fungi associated with crop plants in metal contaminated agriculture fields could be an important step towards reducing agrochemical pollutions. In present study, it was aimed to screen and identify metal resistant endophyte and elucidate its role in rescuing crop plant growth and metabolism during metal stress.

**Results:**

Fungal endophyte, *Penicillium funiculosum* LHL06, was identified to possess higher growth rate in copper (Cu) and cadmium contaminated mediums as compared to other endophytes (*Metarhizium anisopliae, Promicromonospora sp.* and *Exophiala sp.*). *P. funiculosum* had high biosorption potential toward copper as compared to cadmium. An endophyte-metal-plant interaction was assessed by inoculating the host *Glycine max* L. plants with *P. funiculosum* during Cu (100 μM) stress. The Cu application adversely affected the biomass, chlorophyll and total protein content of non-inoculated control plants. The control plants unable to synthesis high carbon, hydrogen and nitrogen because the roots had lower access to phosphorous, potassium, sulphur and calcium during Cu treatment. Conversely, *P. funiculosum-*association significantly increased the plant biomass, root physiology and nutrients uptake to support higher carbon, hydrogen and nitrogen assimilation in shoot. The metal-removal potential of endophyte-inoculated plants was significantly higher than control as the endophyte-association mediated the Cu uptake via roots into shoots. The symbiosis rescued the host-plant growth by minimizing Cu-induced electrolytic leakage and lipid peroxidation while increasing reduces glutathione activities to avoid oxidative stress. *P. funiculosum-*association synthesized higher quantities of proline and glutamate as compared to control. Stress-responsive abscisic acid was significantly down-regulated in the plant-metal-microbe association.

**Conclusion:**

The endophyte *P. funiculosum* symbiosis counteracted the Cu stress and reprogrammed soybean plant growth. Such growth promoting and stress mediating endophytes can be applied at field levels to help in bioremediation of the polluted agricultural fields.

## Background

Heavy metal pollution has become a major issue in the agriculture fields due to recent advancements in industrial and urban activities. Metals like cadmium (Cd) and copper (Cu) are added in the agriculture soil through contaminated irrigated water. Higher concentrations of these metal cause toxicity to living cells, even at very low concentrations. Cadmium can inhibit the growth and yield of plants as a result of chlorosis, instability of lipid membrane, oxidative damage [1] and reaction with functional biochemicals [2-6]. Copper (Cu) is naturally distributed in soil whilst plays essential role in plant growth. It is also important in its function to synthesize enzymes and proteins which are used in various metabolic processes by plants [7]. However, higher concentration is toxic for crops as it interferes with numerous physiological processes [7].

Lack of immobility and solubility of these toxic metals further synergized the negative effects on plant growth. Phytoremediation is argued as a promising method to rehabilitate the polluted soil. However, most of the metal-accumulating plants are not suitable due to their small biomass and slow growth rates. Therefore, it is deemed important to further develop remediation strategies for heavy metal contaminated soils [3,8-10]. In this regard, interactions among metals, microbes and plants have attracted much attention because of the biotechnological potential of microorganisms to remove metals directly from polluted media and the possible role of microorganisms in promoting plant growth in metal contaminated soils [10]. Among microbes, endophytic fungi have been recently known to produce plant growth regulators (like gibberellins and auxins) and extend plant tolerance under abiotic and biotic stresses [11-17]. In addition, it has been shown that endophytic fungi such as *Chaetomium globosum*[18], *Neotyphodium coenophialum*[19] and *Neotyphodium* endophytes [20] have the potential to remove soil contaminants by enhancing phytoremediation potential of the host-plants [21-25].

Soybean growth is often hindered by the calamities of extreme environmental conditions, thus reducing crop yield and growth. Soybean is known to play essential role in human health and food intake specially the East-Asian countries [24]. Previously, we isolated and identified endophytic fungi from the roots-tissues of soybean plants (Table 1). These endophytic fungal species produced gibberellins, increased plant growth and enhanced tolerance against salinity and drought stresses. Endophytes *Penicillium funiculosum, Metarhizium anisopliae,* and *Exophiala sp.* extended greater benefits to host-plants by ameliorating host-physiology during stress conditions. The endophytes were bioactive in promoting *Waito-C* (gibberellins biosynthesis mutant) and Dongjin-beyo (normal gibberellins pathway) rice cultivars. *P. funiculosum* and *Exophiala sp* were detected to produce physiologically active gibberellins through advanced GC/MS selected ion monitor (SIM) techniques [14-16]. *Promicromonospora sp.* was observed to produce Ascotoxin [17] and inhibited the seed germination of lettuce and weed seed. *Metarhizium anisopliae* was inactive to produce gibberellins but played active role to help the host-plants to resist salinity stress [15]. Since these endophytes were active to counteract abiotic stresses and produced phytohormones/secondary metabolites, therefore, we hypothesized that due to this potential they might help the host-plants to avoid heavy metal toxicity. To elucidate plant-microbe-metal interactions, we initially screened four endophytic fungal strains to know their potential to bioaccumulate Cu/Cd in contaminated mediums. The bioactive strain was then associated with host soybean plants to assess the metal removal capacity and influence on the essential biochemicals and plant growth.

**Table 1:** Strains of the fungal endophytes used to determine growth rate under high copper conditions

**Name**	**Plant hormones production**	**Stress condition**	**Identification**	**GeneBank ID**	**References**
*Penicillium funiculosum* LHL06	GA_1,_ GA_4_, GA_8,_ GA_9_	Salinity (70 and 140 mM)	ITS rDNA	HM017065	Khan et al. [15]
			LSU rDNA		
*Metarhizium anisopliae* LHL07	ND	Salinity (70 and 140 mM)	ITS rDNA	HM017066	Khan et al. [16]
			LSU rDNA		
*Exophiala sp* LHL08	GA_1_, GA_3_, A_4_, GA_7_, GA_5_,GA_8_, GA_9_,GA_12_, GA_20_	Salinity and drought	ITS rDNA	HM623425	Khan et al. [14]
			LSU rDNA		
*Promicromonospora sp.* LK1	ND	NST	ITS rDNA	JQ288104	Khan et al. [17]

*GA *(gibberellins); *ITS *(internal transcribed spacer); *LSU* (large subunit); *ND *(not detected); *NST *(no-stress treatments).

## Results

### Screening for metal resistant endophyte

**Figure 1:**
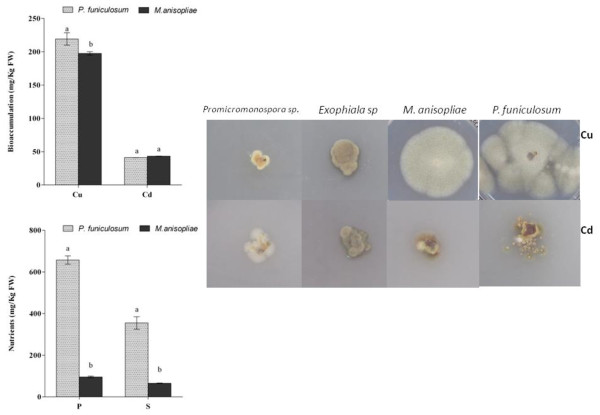
**Bioaccumulation and growth dynamics of endophytic fungi in copper (Cu) and cadmium (Cd) contaminated mediums. **Among four endophytes (*P. funiculosum, M. anisopliae, Promicromonospora sp. *and *Exophiala sp*), only two (*P. funiculosum,* and *M. anisopliae*) had significantly higher growth rates which were selected for metal and nutrient uptake analysis through ICP-MS. For each set of treatment, the different letter indicates significant differences at *P < 0.05 * level by DMRT.

Four different endophytic fungi (*P. funiculosum, M. anisopliae, Promicromonospora sp.* and *Exophiala sp* – Table 1) isolated from soybean plants were screened for their potential to bio-accumulate metal. The results showed that *Promicromonospora sp.* and *Exophiala sp.* did not grow well during ten-days of incubation in Cu and Cd polluted mediums. Conversely, the growth of *P. funiculosum* and *M. anisopliae* was significantly higher during Cu as compared to Cd (Figure 1). The growth area of *Promicromonospora sp., Exophiala sp., M. anisopliae* and *P. funiculosum* was 0.4 ± 0.01, 0.5 ± 0.02, 3.2 ± 0.07 and 5.3 ± 0.09 cm^2^ during Cu stress respectively. In case of Cd stress, the growth of all endophytes was not significantly different from each other. Though the growth area of *M. anisopliae* and *P. funiculosum* was a little higher but it was not significant.

Upon high growth of *M. anisopliae* and *P. funiculosum* in Cu/Cd stress, we carefully removed the mycelial pads and subjected it to ICP-MS analysis to determine the level of metal accumulation. *P. funiculosum* contained significantly higher concentration of Cu as compared to *M. anisopliae* while Cd accumulation was not significantly different between each other (Figure 1). The phosphate and sulphate contents were significantly higher in *P. funiculosum* as compared to *M. anisopliae.* Thus, on the basis of growth dynamics and higher accumulation rate of copper, *P. funiculosum* was selected for further experiments of plant-metal-microbe interactions.

### *P. funiculosum*-association influenced soybean growth and photosynthesis pigments during Cu stress

**Figure 2:**
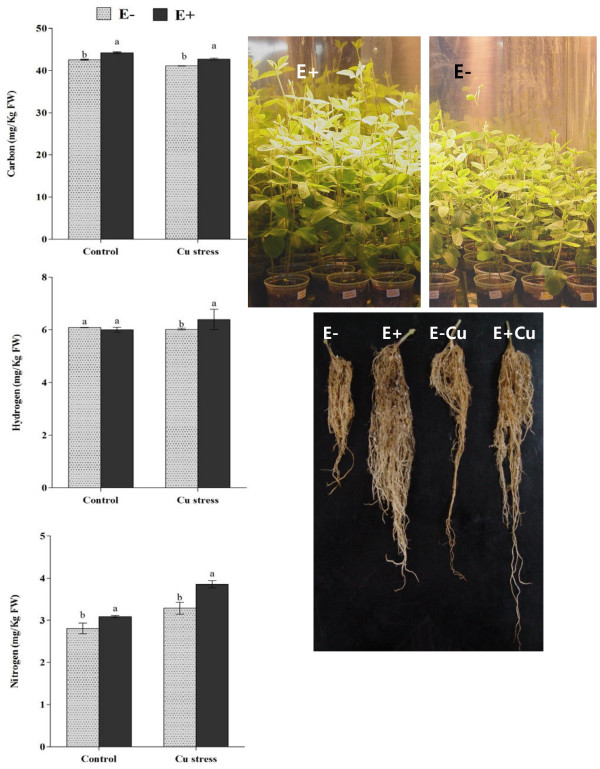
**Effect of copper (Cu) stress on soybean plant growth and essential macronutrient assimilation with (E+) or without (E-) the inoculation of endophyte *P. funiculosum.****‘*E-Cu’ and ‘E + Cu’ refer to plants treated with copper with or without the presence of endophyte respectively. The experiment was replicated three times with three replications each time while each treatment contained 21 plants. For each set of treatment, the different letter indicates significant differences at *P < 0.05 *level by DMRT.

The bioactive *P. funiculosum* was associated with host soybean plants to assess the effect of symbiosis on the plant growth during Cu stress. Endophyte association (E+) with host soybean plants significantly increased the shoot length and biomass as compared to non-inoculated control (E-) plants (Table 2). During Cu stress, the shoot length, shoot and root fresh biomass were considerably reduced in the endophyte-free (E-Cu) plants as compared to *P. funiculosum* infection. The reduced growth of shoot and root was quite visible and significant in E- plants than E + with or without Cu stress (Figure 2). Reduced leaf area and increased leaf curling was significantly higher in E- than E + plants under Cu stress. This enhanced growth was also confirmed from the quantities of essential elements i.e. carbon, hydrogen and nitrogen. The elemental analyses showed that carbon content was significantly higher in the shoot parts of E + plants as compared to E- with or without the excursion of Cu stress (Figure 2). This suggests higher plant biomass assimilation during the symbiosis of endophyte. The hydrogen content was similar in E + and E- plants under normal growth conditions however under Cu stress, it was significantly higher in E + plants than E-. It suggests that the relative proportion of water was significantly higher in the shoot of endophyte-inoculated plants than non-inoculated control plants (Figure 2). Nitrogen assimilation in shoot part of the plants plays a pivotal role in maintaining improved growth and metabolism. During plant-endophyte-metal interaction, the endophyte-inoculated E + plants had assimilated significantly higher level of nitrogen in shoot as compared to control E- plants during Cu stress (Figure 2).

**Table 2:** Effects of endophyte status on copper stress measured by growth of soybean

**Treatments**	**Association**	**Shoot length (cm)**	**Shoot dry weight (g)**	**Root dry weight (g)**
Control	**E-**	31.21 ± 0.64 b	6.30 ±0.14 b	1.10 ±0.01 b
	**E+**	36.17 ± 0.74 a	7.20 ±0.13 a	3.42 ±0.03 a
Copper (100 μM)	**E-**	27.21 ± 0.16 b	6.05 ±0.16 b	1.3 ±0.06 b
	**E+**	33.25 ± 0.51 a	6.96 ± 0.14 a	3.16 ± 0.08 a

Values in the table refer to mean of 21 plants/treatment while ± shows S.E. Means followed by different letter (s) are significantly different (*P* < 0.05) between E- and E + as determined by Duncan’s multiple-range test.

**Figure 3:**
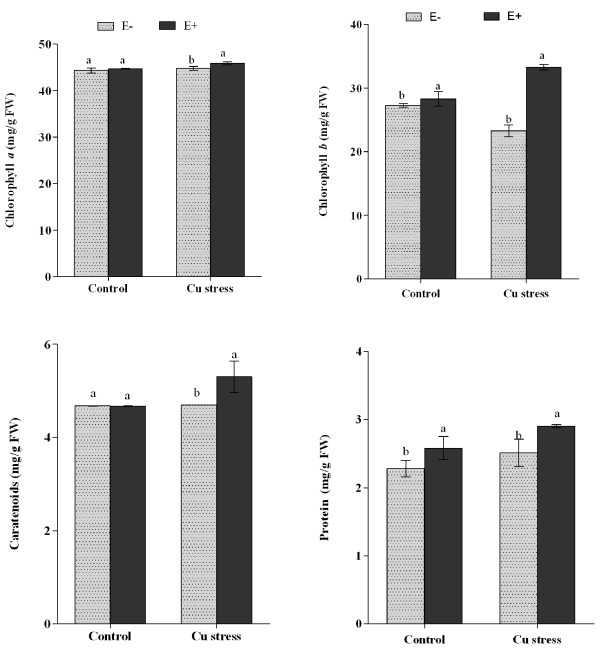
**Influence of copper stress on the chlorophyll, caratenoids and protein contents of soybean plants inoculated with endophyte.***‘*E-Cu’ and ‘E + Cu’ refer to plants treated with copper with or without the presence of endophyte respectively. For each set of treatment, the different letter indicates significant differences at *P < 0.05 * level by DMRT.

To further assess the role of endophyte in improving plant physiology and relieving the adverse effects of Cu stress, photosynthetic pigments like chlorophyll and total carotenoid were determined. The results in Figure 3 showed that during normal growth condition, chlorophyll *a* and total carotenoid content had no significant difference between E- and E + plants however, chlorophyll *b* was significantly higher in E + than E-. Upon Cu stress to soybean plants, the E + plants had significantly higher Chlorophyll *a*, *b* and total carotenoid as compared to E- plants. The increased synthesis of photosynthetic pigments was also confirmed by the level of protein metabolism. Total protein content was significantly higher in E + plants as compared to E- plants under normal growth and Cu stress conditions (Figure 3).

### Copper uptake and root essential nutrients composition

**Figure 4:**
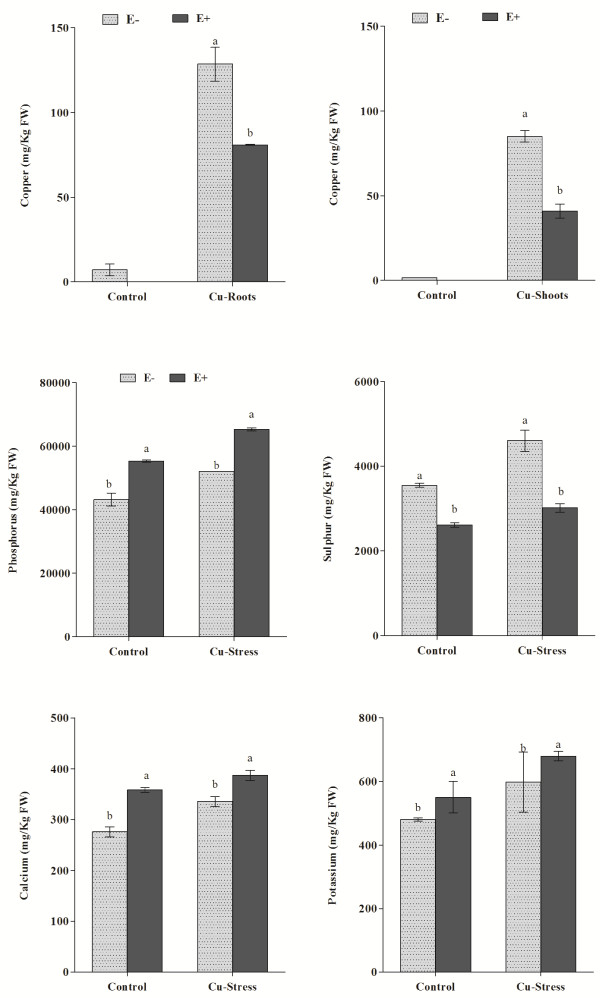
**Accumulation of copper inside soybean roots and its impact on the composition of essential nutrients in association with endophyte (E+). **For each set of treatment, the different letter indicates significant differences at *P < 0.05* level by DMRT.

To elaborate the metal uptake by the soybean roots, the Cu content was assessed through ICP-MS. According to analysis, Cu was not detected in E + plants grown under normal conditions however the E- plants contained a significantly lower amount of Cu in roots. Under Cu stress, a significantly higher Cu uptake was observed in the non-inoculated control plants (E-) as compared to *P. funiculosum-*inoculated plants (E+; Figure 4). A similar trend was also revealed in the Cu accumulation in soybean shoots. The E- plants had significantly higher Cu content in their shoots as compared to E + during Cu-stress (Figure 4). Furthermore, there was a significant difference between E + and E- to remove Cu. From the Cu quantification, it was also observed that the root parts had higher concentration of Cu as compared to shoot which suggest endophytic intervention of Cu transport into shoots via roots (Figure 4). This was also confirmed from the Cu removal capacity of endophyte-inoculated plants. The result showed that the Cu tolerance rate was significantly higher in E + (81.3%) as compared to E- (43.4%). The transport index was also significantly different between E + and E-. The *P. funiculosum* inoculated plants had lower Cu transport rate (37.8%) as compared to E- (66.1%). It indicates that endophytic fungal-association has mitigated the Cu induced abiotic stress.

Essential nutrients i.e. sulfur and phosphorus uptake from the soil by roots was significantly higher in E + plants as compared to E- plants. Phosphorus (P) is a major nutrient for plant growth and reproduction. Phosphorus content was significantly higher in the roots of E + plants under normal and Cu stress conditions as compared to E- plants (Figure 4). Current findings suggest that *P. funiculosum* helped the soybean plants in the availability of P even during stress conditions. Sulfur, being taken from soil into plant tissues, plays essential role in building parts of plant proteins and important amino acids. Significantly higher contents of sulfur was found in the roots tissues of *P. funiculosum*-associated plants as compared to non-inoculated control plants under normal and Cu stress conditions (Figure 4). Calcium (Ca) in plant’s roots plays a pivotal role in plant growth and response to abiotic stress and microbial association [26]. Our results showed that endophyte *P. funiculosum* association significantly activated Ca signaling in the roots of soybean plant as compared to control (Figure 4). The same trend was also observed under Cu stress. Potassium (K) content was also significantly higher in E + plants than E- plants under normal and Cu stress conditions.

### Effects on electrolytes, lipid peroxidation and reduced glutathione

**Figure 5:**
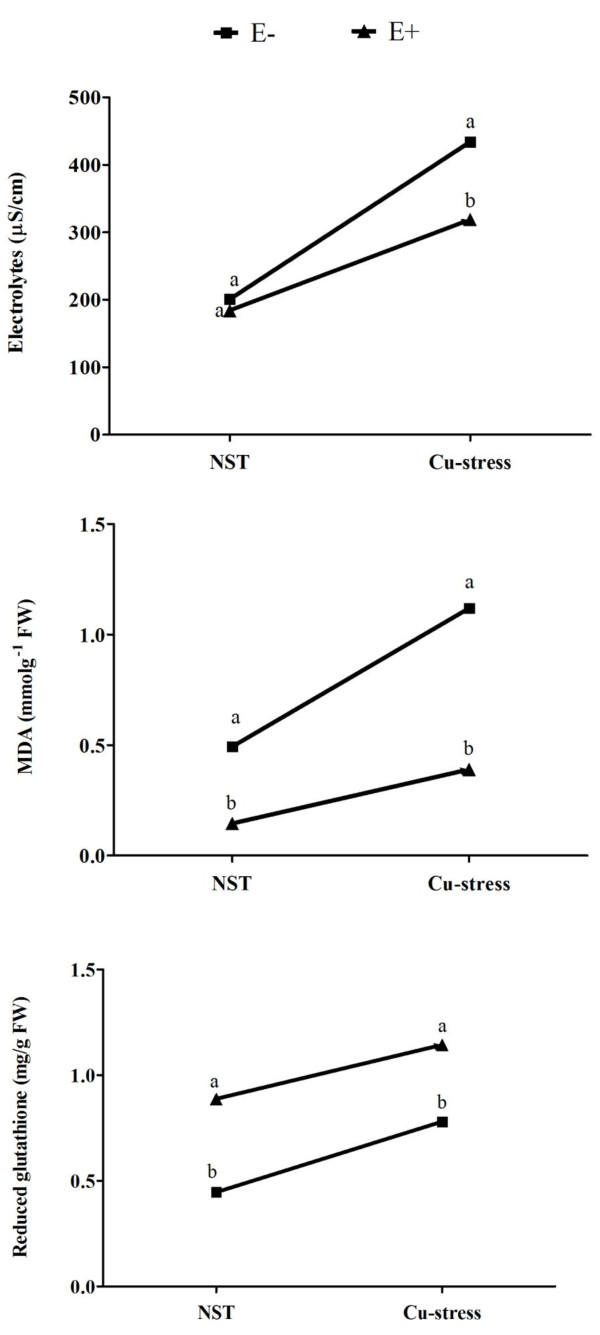
**Effect of Cu stress on the membrane damage (electrolytes and lipid peroxidation) and oxidative stress (reduced glutathione) of soybean plants associated with (E+) or without (E-) endophyte. **NST refers to no-stress treatment/normal growth condition. For each set of treatment, the different letter indicates significant differences at *P < 0.05 *level by DMRT.

The electrolytes release from plant tissues was not significantly different between E + and E- plant under normal conditions. However, when soybean plants were exposed to Cu stress, the rate of electrolytes formation was significantly higher in non-infected soybean plants as compared to *P. funiculosum* infected plants (Figure 5). The results suggest that endophyte-association reduced the functional membrane damage under Cu stress. This was also in conformity with lipid peroxidation of leave tissues of endophyte-associated plants. It is known that with excursion of stress conditions the peroxides of polyunsaturated fatty acids generates malondialdehyde (MDA) which is the most abundant individual aldehydic lipid-layer breakdown product [27]. Normally abiotic stress induces the rate of lipid peroxidation by producing higher amount of MDA. However, this trend was minimized by the endophyte-symbiosis with soybean plants as compared to control plants during Cu stress (Figure 5). Additionally, the Cu toxicity also develops oxidative stress inside plant tissues which is counteracted by the recruitment of antioxidants like glutathione. Reduced glutathione (GSH) was extensively synthesized in endophyte-inoculated plants than the non-inoculated ones under Cu stress and normal growth conditions (Figure 5).

### Regulation of free amino acid and abscisic acid in host-plants under Cu stress

**Figure 6:**
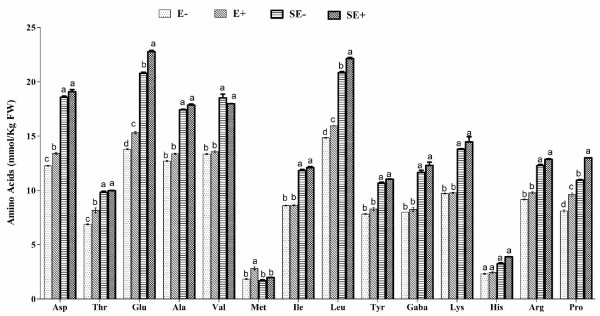
**Free amino acid accumulation in soybean roots associated with endophyte free and infected 3-week-old plants following a ten day copper stress treatment. **For each set of treatment, the different letter indicates significant differences between E- and E + at *P < 0.05 *level by DMRT. SE- (plants without endophyte and Cu stress) and SE + (plants with endophyte-inoculation and Cu stress).

Plants synthesize its amino acids using nitrogen and related intermediaries obtained through roots from the soil [28]. The free amino acid synthesis was higher during endophyte-association under normal growth conditions as compared to endophyte-free counterpart. The aspartic acid (Asp), threonine (Thr), glutamine (Glu), leucine (Leu), methionine (Met) and proline (Pro) were significantly higher in E + plants than E- under normal conditions (Figure 6). The other amino acids were either similar or in-significantly higher in E + than E- plants. Free amino acid production was further stimulated by Cu stress in the presence of endophyte (SE+). The level of Pro, Glu, and Leu was significantly higher in *P. funiculosum* associated plants as compared to non-infected (SE-) plants.

**Figure 7:**
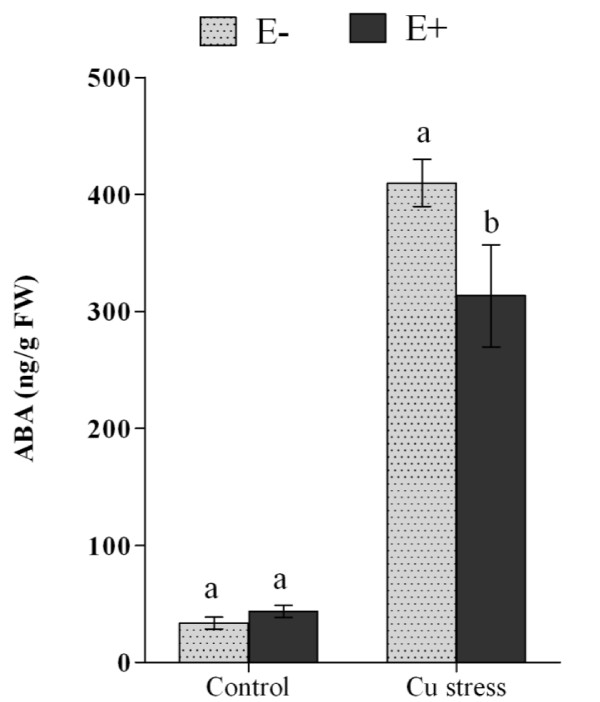
**Effect of stress responsive endogenous abscisic acid (ABA) content of soybean root under Cu stress in association with (E+) or without (E-) endophyte. **For each set of treatment, the different letter indicates significant differences between E- and E + at *P < 0.05 *level by DMRT.

Endogenous stress-responsive phytohormone abscisic acid (ABA) was accumulated upon exposure to abiotic stresses including heavy metal. The control plants (E-) when treated with Cu accumulated significantly higher level of ABA. Conversely, when endophyte-infected plants were treated with Cu, the ABA level was observed significantly lower as compared to non-inoculated control plants (Figure 7).

## Discussion

In the present study, bioactive endophytic fungal strains were initially screened for their growth potential in Cd and Cu polluted mediums. The results suggested that *P. funiculosum* had higher growth rate as compared to the other three *M. anisopliae, Promicromonospora sp.* and *Exophiala sp* endophytes. *P. funiculosum* had higher potential to dwell in excessive Cu than Cd. The role of endophytic fungi has been recently elucidate by Li et al. [23] however, this more concentrate on the bacterial strains than fungal strains. Few endophytes like *Microsphaeropsis, Mucor, Phoma, Alternaria, Peyronellaea, Steganosporium,* and *Aspergillus* have been known to grow well in polluted medium and protect plants from adverse effects of metal stress [23]. Very little has been known about endophytic *Penicillium* and its role in host-plant resistant to metal stress. Some strains of *Penicillium janthinellum* and *P. simplicissimum* have been found to grow well in high Cu medium [29], suggesting that they bears higher biosorption capacity against Cu. Previous studies showed that some strains of *Penicillium* can extend tolerance to host plants against metal toxicity. For example, strains of *P. janthinellum* and *P. simplicissimum* reduced the aluminum and zinc toxicity as it produced citric acid [30]. Similarly, an isolate of *Penicillium* sp. bioaccumulated Cd during incubation period [31]. The findings of studies suggest that strains/species of *Penicillium* can mitigate the Cd and other metal-related toxicity, which can be attributed to their potential to produce bioactive metabolites or enzymes [30,31]. Previously, it was noted that *P. funiculosum* produce bioactive gibberellins which can contribute to the ability of a fungus to convert the toxic metal into stable complexes [31]. A similar effect of stabilizing the negative effects of sodium chloride toxicity was also observed when this endophyte was inoculated to soybean plants [15].

Association of endophytic fungi with crop plant can increase plant fitness under abiotic stress conditions [11,13,32]. The secretion of plant growth regulating compounds by the endophyte probably is the mechanism responsible for the enhancement of plant growth [11,13]. Since the culture filtrate of *P. funiculosum* showed the presence of physiologically active gibberellins [15], therefore, we inoculated soybean plants with the culture medium including propagules of the endophyte. Application of such phytohormones producing endophytes can counteract adverse effects of abiotic stresses on plant growth as shown by Khan et al. [15]. Indeed the fungal associations have ameliorated the growth of Arabidopsis [8], Rapes [10], *Solanum nigrum*[22], *Festuca arundinacea* and *Festuca pratensis*[6], *Lolium arundinaceum*[21] under various Cd/Ni stresses. Cu has been known to induce reduction in photosynthesis, water and nutrient uptake. Plants grown in soil containing high levels of Cu show visible symptoms of injury reflected in terms of chlorosis, growth inhibition, browning of root tips, and finally death [33]. In addition, Cd causes decreased stomatal conductance [34] which affect photosynthetic rates. In present study, we observed that endophytic-fungal association activated the growth of soybean plants by improving plant biomass and synthesizing high chlorophyll, carotenoid and protein to counteract the Cu stress. The ameliorative impacts of endophyte-association were also rectified by the shoot’s carbon and hydrogen content after excursion of Cu stress. A similar effect was also observed when *Neotyphodium* endophytes were colonized to various grass species. Its symbiosis protected the host plants from metal toxicity of aluminum [19], cupper [20] and zinc [35].

In addition to plant biomass loss, excessive Cu pollution also reduces the availability and uptake of essential micronutrients in both root and shoot [4]. Conversely in present results, the endophyte-infection increased the K, Ca and P contents in root tissues as compared to control (E-) plants under Cu stress. Besides potassium’s role in plant cellular metabolism, it plays essential part in oxidative stress responses by helping the synthesis of glutathione (GSH) which is associated with stress tolerance [36]. In present study, the GSH level was significantly high in E + plants than E- under Cu stress. These results suggest that copper alters the equilibrium between synthesis and utilization of GSH either due to its antioxidant role or by serving as a precursor in the synthesis of phytochelatins [36]. Similarly, our results showed that Ca content was high in the presence of endophyte during stress. Ca has an essential role in plant growth and signal transduction related to many biotic and abiotic circumstances [26,37]. To minimize Cu toxicity and rescue plant productivity, it is essential for plants to uptake freely available mineral nutrients (such as Ca). This also indicates that increased endophytic-fungal colonization with soybean plants resulted in activation of Ca signaling to counteract stress. Previously, Kováčik et al. [4] indicated activation of Ca upon Cu stress. Among nutrients, phosphate is major macronutrient for plants to sustain cellular metabolism whilst availability of free phosphate can extend higher growth and metabolism in host plants [38-40]. Microorganisms are major contributor in this function. Previously, we found that *P. funiculosum* had high phosphate (P) solubilization potential [15] and hence in present study, the same helped the host soybean plants to accumulate high P as compared to endophyte-free plants during Cu stress. These ameliorative effects on soybean plants were also rectified by the reduced levels of Cu accumulation in the roots of E + plants as compared to E- plants. It further suggests that endophyte might have higher sorption of Cu during association with host-soybean plants thus accumulating low level of Cu inside roots. However, the in-depth mechanism is still unclear.

It is evident that Cu toxicity to plants also results in membrane damage due to generation of reactive oxygen species. During stress, the malondialdehyde (MDA) is generated which indicates impact on plant membrane [27]. The significantly higher level of MDA in E- plants shows injury to functional membrane during Cu stress. This was also confirmed by the high electrolytic leakage of E- plants. However, during symbiosis with endophyte, most of the adverse effects of Cu toxicity were minimized which suggests the ameliorative function of endophyte. Excessive Cu toxicity, on the other hand, adversely affect the physiologically important free amino acid metabolism [41] because it lies at the crossroad between nitrogen assimilation, carbon fixation and secondary metabolism [28,42]. Current results showed reduced amino acid metabolism in E- plants as compared to E + plants during Cu stress, which suggest rescuing role of endophyte-association to enhance cellular metabolism and improve plant growth. E + plants had significantly higher Pro, Glu, and Leu under Cu stress. Proline (Pro) has been widely known to act as osmo-protectant during abiotic stress condition whilst its accumulation in E + plants showed restoration of growth against stress. Current findings of higher nitrogen assimilation by E + plants further corroborate with the fact that even in stress conditions, the glutamate cycle and amino acid inter-conversion were not significantly affected as compared to E- plants.

Heavy metals such as Cu toxicity to the plants also increase the biosynthesis of endogenous abscisic acid (ABA) (see review of Tuteja [43] and reference therein). Higher ABA may also inhibit the photosynthesis rate as stress perception causes the closure of stomata to avoid dehydration. This further reduces leaf area and plant shoot length with the passage of stress period just like E- plants under stress. In present study, ABA level was significantly lower in E + plants as compared to E- plants under Cu stress. While after ten days of stress period, the E + plants still maintained higher growth as was noticed during the quantification of free amino acids, essential nutrients and ABA. Previous studies suggest that fungal inoculation can increase ABA content in leaves and roots as compared to non-inoculation control plants [43-46]. Contrarily, current findings showed that endophyte-associated soybean plants had reduced ABA as compared to control plants during stress. This altered level of ABA confirms the findings of Mauch-Mani and Mauch [45] and Jahromi et al., [46], who observed low level of ABA under stress and fungal-association. This could be due the gibberellins secretion potential of the endophytes because the non-gibberellins producing strains did not contributed towards the bioaccumulation of Cd/Cu during the screening experiment. A similar behavior of endophytes was also observed in some of the previous studies [13-15]. Sharp et al. [47] reported similar results with *flacca* mutant, however, in present study, the phosphate solubilization and gibberellins production potential of endophyte *P. funiculosum* has not only reprogram the plant growth but also helped the soybean plants to tolerate Cu stress.

## Conclusion

The findings of the present study suggest that endophytes such as *P. funiculosum* can not only improve plant biomass but also resist to the toxic effects of metal contamination. Such beneficial impacts are due to their potential to secrete bioactive gibberellins as noted previously which might help in converting the metal into stable complexes. This could be also attributed to the reduced level of metal inside roots and shoot of soybean plants in symbiosis with endophyte. Influencing soybean plant’s root physiology whilst delineating ameliorative impacts on shoot growth is an ideal strategy to achieve the dual uses of increased crop productivity as well as reduced toxic effects of copper pollution. However, it is also essential to understand the *in vivo* mechanisms by which the gibberellins producing endophytes reduce the metal toxicity.

## Methods

### Endophyte growth and heavy metal resistant strain

*P. funiculosum, M. anisopliae, Promicromonospora sp.* and *Exophiala sp* (Table 1) were isolated from the roots of soybean/cucumber plants and were identified through DNA extraction, PCR techniques, sequencing and phylogenetic analysis of Internal Transcribed Spacer (ITS) and Large Sub-unit (LSU) rDNA. The universal primers used for this purpose were: ITS-1 (5’-TCC GTA GGT GAA CCT GCG G-3’) and ITS-4 (5’-TCC TCC GCT TAT TGA TAT GC-3’) and LR0R (F) (5′-ACC CGC TGA ACT TA AGC-3′) and TW13(R) (5′-GGT CCG TGT TTC AAG ACG-3′) as described by Redman et al. [13].

The isolated strains were grown in potato dextrose agar (PDA) having 50 μM concentration of cadmium [Cd_3_(PO_4_)_2_] and copper (CuSO_4_) respectively. After 10 days of incubation (28°C in darkness), the growth rate of endophytes was measured as describe by Pan et al. [25] and Alhamed and Shebany [18]. Each treatment had five replications to assess the growth zone development. On the basis of results, the bioactive strain was grown in the Czapek broth (500 ml containing 1% glucose, 1% peptone, 0.05% KCl, 0.05% MgSO_4_.7H_2_O, and 0.001% FeSO_4_.7H_2_O; pH 7.3 ± 0.2) and incubated for 10 days at 30°C under shaking conditions (120 rpm).

### Plant growth and heavy metal stress

Soybean (*Glycine max* L. var. hwangkeumkong) seeds (moisture content 6%; germination 85%) were donated by Prof. Dong-Jang Lee (School of Applied Biosciences, Kyungpook National University, Republic of Korea). Seeds were surface-sterilized by immersing in 20% ethanol for 30 sec and then in 0.2% solution of Hg_2_Cl_2_ for 2 min, followed by several rinses with sterile double distilled water to remove disinfectant [48]. The sterilized seeds were incubated (28°C and relative humidity of 60%) for 24 h to obtain an equal germination (moisture content 78.1% at 26°C in darkness). To assess the interaction of plant-microbe- metal, the germinated seeds were sown in autoclaved pots (30 × 15 cm; 1 Kg/pot at 121°C for 15 min) containing the substrate composed of: peat moss (10-15%), perlite (35-40%), coco-peat (45-50%) and zeolite (6-8%) with macro-nutrients present as: NH_4_ ~0.09 mg.g^-1^; NO_3_ ~0.205 mg.g^-1^; P_2_O_5_ ~0.35 mg.g^-1^and K_2_O ~0.1 mg.g^-1^[49].

The heavy metal-resistant endophytic strain, grown in Czapek broth (20 ml for each pot containing 2–3 g of fungal propagules) was added to the pots to initiate the infection process of soybean roots. The control plants only received endophyte-free Czapek broth (20 ml/pot) using the same recipe and conditions as mention in previous section. Thus, soybean plants and endophyte were grown together for three weeks in the growth chamber (day/night cycle: 14 h; 28°C/10 h; 25°C; relative humidity 60–70%; light intensity 1000 μEm^-2-^s Natrium lamps) and irrigated with distilled water. The experimental designed included endophyte-free control plants with or without metal stress and endophyte-infected plants with or without metal stress. Three weeks grown soybean plants with and without endophytic fungal association were applied with copper stress (CuSO_4_) 100 μM for 10 days. The Cu solution (100 ml per pot i.e. 16 mg/Kg Cu in each pot filled with 1 Kg autoclaved soil) was applied after every 24 h for 10 days. Each time plants were irrigated before Cu treatment to avoid leaching. Thus, the final concentration of Cu remained the same throughout the experiment. The experiment was replicated three times with three replications each time while each treatment contained 21 plants.

After stress period, the shoot length was measured while the shoot and root biomass of soybean plants were measured by drying them in oven at 65°C for 72 h. For photosynthetic pigments, lipid peroxidation and antioxidant, fresh plants samples were used while for elemental/metal and plant hormonal analysis, plant samples were freeze dried for 4–5 days (Virtis Freeze Dryer, Gardiner, NY, USA). All the readings were taken in triplicate. To determine the plant translocation ability of metal via root to shoot, the transport index [(Cu shoot mg Kg^-1^**/**Cu root mg Kg^-1^) × 100] was calculated as suggested by Soleimani et al. [6]. While the Cu removal or tolerance capacity was measured through this formula:

$$\left[\frac{\mathrm{Cu}\;\text{shoot}\;\times \;\text{shoot}\;\text{weight}\;+\;\mathrm{Cu}\;\text{root}\;\times \;\text{root}\;\text{weight}}{\text{Total}\;\mathrm{Cu}\;\text{added}/\text{plot}}\right]\times 100$$

### Determination of photosynthetic pigments

Photosynthetic pigments were extracted from leaves of soybean plants ground with 80% acetone. The chlorophylls and carotenoid were estimated according to the method of Lichtenthaler [50]. The absorbance for chlorophyll ***a*** and ***b*** and carotenoid was recorded at 663, 645 and 470 nm, respectively. Chlorophyll content was calculated using the following formulae:

$$\begin{array}{l} •\;\text{Chlorophyll}\;a\left(\mathrm{mg}/g\;\mathrm{FW}\right)\\ \;\;=[\{\left(12.7\;\times \;A_{663}\right)–\left(2.69\;\times \;A_{645}\right)\}/1,000\;\times \;W]\mathrm{xV}\\ •\;\text{Chlorophyll}\;b\left(\mathrm{mg}/g\;\mathrm{FW}\right)\\ \;\;=[\{\left(22.9\;\times \;A_{645}\right)–\left(4.68\;\times \;A_{663}\right)\}/1,000\;\times \;W]\;\mathrm{xV}\\ •\;\text{Total}\;\text{carotenoid}\;\text{content}\\ \;=\left({1,000\;A}_{470}–1.82\;\text{chlorophyll}\;a–85.02\;\text{chlorophyll}\;b\right)/198 \end{array}$$

Where *W* is the fresh weight and *V* is the extraction volume.

### Determination of electrolytes, lipid peroxidation and reduced glutathione

Leaf electrolyte (E) was determined as reported by González and González-Vilar [51]. Fresh leaves (200 mg) samples were cut into 5 mm length and placed in test tubes containing 10 ml deionized distilled water. The tubes covered with plastic caps were placed in a water bath at a constant temperature of 30°C. After 2 h the initial electrical conductivity (EC_1_) was measured using an electrical conductivity meter. The samples were kept at 121°C for 20 min to completely kill the tissues and release all electrolytes. The samples were then cooled to 25°C and final electrical conductivity (EC_2_) was measured. The electrolytes released were estimated using formula: E = EC_1_/EC_2_. The experiment was repeated three times and results were expressed in μS/cm.

To confirm the electrolytes released and damages to the plant membrane after Cu toxicity, extent of lipid peroxidation was determined by the method of Ohkawa et al. [52]. For this assay, 0.2 ml 8.1% sodium dodecyl sulphate, 1.5 ml 20% acetic acid (pH 3.5) and 1.5 ml 0.81% thiobarbituric acid aqueous solution were added in succession in a reaction tube. Then 0.2 ml tissue homogenate extracted with 10 mM phosphate buffer (pH 7.0) was added. The mixture was heated in boiling water for 60 min. After cooling to room temperature, 5 ml butanol: pyridine (15:1 v/v) were added. The upper organic layer was separated and the optical density of the resulting pink color was recorded at 532 nm using spectrophotometer. Tetramethoxypropane was used as an external standard. The level of lipid peroxides was expressed as micro moles of malondialdehyde (MDA) formed/g tissue weight. The experiments were repeated three times.

To determine reduced glutathione (GSH), fresh leaves tissues (100 mg) were ground in 3 ml 5% (v/v) trichloroacetic acid using chilled mortar and pestle. The homogenates were centrifuged at 2,500 *× g* for 15 min at −4°C. The supernatants were analyzed for the content of reduced glutathione (GSH) according to the method of Ellman [53]. Supernatant (0.1 ml) was added to 3.0 ml 150 mM NaH_2_PO_4_ (pH 7.4) containing 0.5 ml of Ellman’s reagent. The mixture was incubated at 30°C for 5 min. Absorbance was determined at 412 nm and the GSH concentration was calculated by a standard curve.

### Determination of Cu and essential nutrients uptake

In the screening experiment of metal and endophyte, the grown endophyte pads were detached from PDA plates and subjected to Inductively Coupled Plasma Mass Spectrometry (ICP-MS; Optima 7900DV, Perkin-Elmer, USA) analysis of Cd/Cu to differentiate and identify higher metal accumulating strain [54]. Similarly, after metal-plant-microbe interaction, the metal-treated soybean roots and shoot associated with or without endophytes were assessed for metal uptake and its influence on the accumulation of essential nutrients. The plant samples were immediately shifted into liquid nitrogen and freeze dried (−55°C; Virtis Freeze Dryer, Gardiner, USA) for 3–4 days. The contents of Cu and essential nutrient like phosphorus, sulfur, potassium and calcium were determined by ICP-MS while carbon, hydrogen and nitrogen contents in the shoot parts were determined by the elemental analyzer (Flash2000, ThermoFisher Scientific Inc., Waltham, MA, USA).

### Free amino acid extraction and quantification

All the treated soybean plants roots were immediately shifted to liquid nitrogen and then kept at −80°C. The roots parts were freeze dried (−55°C; Virtis Freeze Dryer, Gardiner, NY, USA). The root samples (100 mg) were extracted with 2 ml 10% trichloroacetic acid with gentle agitation on a shaker (110°C for 24 h; 50 rpm). The filtrate was dried and dissolved in 0.02 N HCl to obtain supernatant through centrifugation (10,000 rpm for 15 min). The amino acid compositions were then obtained by automatic analysis algorithm of the amino acid automatic analyzer. The amino acid analyzer (HITACHI L-8900, Japan) attached to HITACHI HPLC (packed column with ion-exchanging resin – No. 2622 PF; 4.6 × 60 mm) and UV detector (VIS1: 570 nm, VIS2: 440 nm) was used for analysis of free amino acids. Wako L-8500 buffer solution PF-1, 2, 3, 4 and RG were used as mobile phase. About 20 μl of each sample was injected. Free amino acids were determined by using Ninhydrin reagent set (Wako Chemical Inc, Japan). All samples were run in triplicates and expressed in mmol/Kg fresh weight.

### Abscisic acid extraction and quantification

The endogenous abscisic acid (ABA) content was quantified from the freeze dried samples by following the protocols of Kamboj et al. [55]. Plant samples were extracted with 30 ml of extraction solution containing 95% isopropanol, 5% glacial acetic acid, and 20 ng of Me-[^2^H_6_]-ABA. The filtrate was concentrated by a rotary evaporator. The residue was dissolved in 4 ml of 1 N sodium hydroxide solution, and then washed three times with 3 ml of methylene chloride to remove lipophilic materials. The aqueous phase was brought to approximately pH 3.5 with 6 N hydrochloric acid and partitioned three times into ethyl acetate (EtOAc). EtOAc extracts were then combined and evaporated. The dried residue was dissolved in phosphate buffer (pH 8.0) and then run through a polyvinylpolypyrrolidone (PVPP) column. The phosphate buffer was adjusted to pH 3.5 with 6 N HCl and partitioned three times into EtOAc. EtOAc extracts were combined again and evaporated. The residue was dissolved in dichloromethane (CH_2_Cl_2_), and passed through a silica cartridge (Sep-Pak; Water Associates, Milford, Massachusetts, USA) which was pre-washed with 10 ml of diethyl ether: methanol (3:2, v/v) and 10 ml of dichloromethane. ABA was recovered from the cartridge by elution with 10 ml of diethyl ether (CH_3_-CH_2_)_2_O: methanol (MeOH) (3:2, v/v). The extracts were dried and methylated by adding diazomethane for GC/MS-SIM (6890 N network GC system, and 5973 network mass selective detector; Agilent Technologies, Palo Alto, CA, USA) Additional file 1: Table S1 analysis. For quantification, the Lab-Base (ThermoQuset, Manchester, UK) data system software was used to monitor responses to ions of m/e 162 and 190 for Me-ABA and 166 and 194 for Me-[^2^H_6_]-ABA.

### Statistical analysis

To identify significant effects between the treatments and control with or without stress conditions and endophyte, Duncan’s multiple range tests (DMRT) was adopted by using Statistic Analysis System (SAS 9.1, USA) (*P* < 0.05). The mean, standard error and the graphical representation was done through Graph Pad Prism software (version 5.0, San Diego, California USA).

## Competing interests

The authors declare that they have no competing interest.

## Authors’ contribution

ALK planned, designed and conducted the experiments. IJL performed phytohormonal quantification and amino acid analysis. ALK and IJL both contributed in writing this manuscript. Both authors read and approved the final manuscript.

## Supplementary Material

Additional file 1: Table S1 GC-MS conditions used for analysis and quantification of the plant endogenous ABA. Click here for file
