# Supplementary material for: Endophytic Penicillium funiculosum LHL06 secretes gibberellin that reprograms Glycine max L. growth during copper stress
Source: BMC Plant Biol. 2013 May 31;13:86. doi: 10.1186/1471-2229-13-86 (PMC3674946; doi:10.1186/1471-2229-13-86)
Supplement: Additional file 1: Table S1 — GC-MS conditions used for analysis and quantification of the plant endogenous ABA. [file 1471-2229-13-86-S1.doc]

**Additional file 1: Table S1**

GC-MS conditions used for analysis and quantification of the plant endogenous ABA

| **Equipment** | **Hewlett-Packard 6890, 5973N Mass Selective Detector** |
| --- | --- |
| **Column** | HP-1 capillary column  (30m×0.25㎜ i.d. 0.25㎛ film thickness) |
| **Carrier gas** | He (40 ㎖/min.) |
| **Source temperature** | 250℃ |
| **Oven conditions** | ABA: 60℃(1 min.)→15℃/min.→200℃→5℃/min.→  250℃→10℃/min.→280℃ |
| **Injector temperature** | 200℃ |
| **Ionizing voltage** | 70 ev |
